# Oxidoreductases in Glycoprotein Glycosylation, Folding, and ERAD

**DOI:** 10.3390/cells9092138

**Published:** 2020-09-22

**Authors:** Chaitanya Patel, Haddas Saad, Marina Shenkman, Gerardo Z. Lederkremer

**Affiliations:** 1 The Shmunis School of Biomedicine and Cancer Research, Cell Biology Division, George Wise Faculty of Life Sciences, Tel Aviv University, Tel Aviv 69978, Israel; chaitanyspatel@gmail.com (C.P.); s.haddas@gmail.com (H.S.); shenk@tauex.tau.ac.il (M.S.); 2 Sagol School of Neuroscience, Tel Aviv University, Tel Aviv 69978, Israel

**Keywords:** PDI, oligosaccharyltransferase, calnexin, ER quality control, mannosidase, ERAD

## Abstract

N-linked glycosylation and sugar chain processing, as well as disulfide bond formation, are among the most common post-translational protein modifications taking place in the endoplasmic reticulum (ER). They are essential modifications that are required for membrane and secretory proteins to achieve their correct folding and native structure. Several oxidoreductases responsible for disulfide bond formation, isomerization, and reduction have been shown to form stable, functional complexes with enzymes and chaperones that are involved in the initial addition of an N-glycan and in folding and quality control of the glycoproteins. Some of these oxidoreductases are selenoproteins. Recent studies also implicate glycan machinery–oxidoreductase complexes in the recognition and processing of misfolded glycoproteins and their reduction and targeting to ER-associated degradation. This review focuses on the intriguing cooperation between the glycoprotein-specific cell machineries and ER oxidoreductases, and highlights open questions regarding the functions of many members of this large family.

## 1. Introduction

Secretory and membrane proteins are folded in the endoplasmic reticulum (ER) with the aid of various molecular chaperones and oxidoreductases, which catalyze oxidation and reduction reactions on their cysteine residues. The great majority of the secretory proteins are glycosylated, mostly by addition of one or more N-linked oligosaccharides covalently attached to asparagine (Asn) sidechains. Nascent N-linked glycoproteins possess a large oligosaccharide precursor, Glc_3_Man_9_GlcNAc_2_, which is later sequentially trimmed. Protein folding and acquisition of the correct native structure depends on the addition and proper processing of these sugar chains. Defects in N-linked glycosylation can cause a number of inherited diseases named congenital disorders of glycosylation. The ability of mammalian cells to correctly identify and degrade misfolded secretory proteins is crucial for their correct function and survival. An inefficient disposal mechanism results in the accumulation of misfolded proteins and consequent ER stress.

Disulfide bond formation is one of the most important modifications affecting secretory protein folding, which is catalyzed by the disulfide isomerase (PDI) family of proteins. Most glycoproteins have disulfides, which are present in 78% of the secretory proteins [1]. The oxidative environment in the ER enables ER-resident oxidoreductases to facilitate disulfide bond formation, which stabilizes protein structures. Proper redox conditions in the ER, together with regulation of calcium homeostasis, are vital for the functions of many luminal pathways and the maintenance of homeostasis.

Oxidoreductases are enzymes involved in the protein folding process, which are responsible for modifying exposed cysteine thiol groups by oxidation, reduction, and isomerization. The oxidoreductases contain domains structurally similar to cytosolic thioredoxin, called thioredoxin-like domains. The reaction of disulfide bond formation is assisted by exchange of two electrons between the cysteine of the substrate protein and cysteines in a catalytic Cys-X-X-Cys motif in the thioredoxin-like domain of the oxidoreductases (reviewed by Robinson and Bulleid in this Special Issue [2]). Over 20 oxidoreductases have been identified in the ER, of which PDI, also known as P4HB/PDIA1, is the best characterized. Some of these enzymes contain more than one thioredoxin-like domain, including some that are not catalytic (e.g., PDI contains two active and two non-catalytic thioredoxin-like domains). The enzymes are soluble ER luminal proteins, except for the five members of the TMX subfamily, which have a transmembrane domain (reviewed by Guerra and Molinari in this Special Issue [3]). The oxidoreductases catalyze disulfide bond formation by oxidizing the cysteine residues of the folding protein. The reduced oxidoreductase molecules are then re-oxidized by several oxidases—Ero1α, Ero1β, Prx4, GPX7, and GPX8 [4,5,6,7,8]. Ero1α is the main oxidase involved, transferring a pair of electrons to molecular oxygen in a flavin-mediated reaction to yield hydrogen peroxide. Several of the oxidoreductases have the capability, and in some cases a preference, to reduce disulfide bonds. The stability of the active-site disulfides determines the function of the PDI family member in reduction or oxidation. Comprehensive reviews of the oxidoreductases and their mechanism of action can be found in [9,10,11].

There is also a series of ER-resident oxidoreductases that are selenoproteins. Selenoproteins are proteins that incorporate an atom of selenium instead of sulfur in one or more cysteines, which are then called selenocysteine, abbreviated as Sec (U). Incorporation of Sec into a selenoprotein is a result of transfer from a selenocysteine-specific tRNA, which decodes an UGA codon in the mRNA. Selenoproteins intervene in numerous physiologic pathways, such as immune responses, antioxidant defense, redox signaling, and thyroid hormone metabolism. Some of the selenoproteins function as oxidoreductases, containing thioredoxin-like domains with one or more selenocysteines instead of cysteine in a catalytic Cys-X-X-Sec or similar motif [12,13,14].

In addition to their catalytic activity in disulfide bonding, many of the oxidoreductases were shown to recognize hydrophobic pockets in their substrate proteins, and thus they are in fact folding sensors. They participate in the recognition of misfolded glycoprotein domains during sugar chain trimming and creation of what was termed the glycan code for targeting to ER-associated degradation (ERAD) [15]. The oxidoreductases escort and functionally interact with glycoproteins, from their translocation into the ER and glycosylation to late stages of productive folding or targeting to ERAD. Several oxidoreductases have been found in recent years to associate functionally with the glycan recognition and processing machineries in the ER, which is the focus of this review.

## 2. Oxidoreductases in the Oligosaccharyltransferase Complex

The majority of secretory proteins are N-glycosylated, a reaction that occurs mostly co-translationally in higher eukaryotes. In the ER lumen, the translocating proteins are exposed to a variety of enzymes and chaperones that promote protein maturation through both folding and post-translational modifications. At the luminal side of the Sec61 translocation channel, the proteins encounter a membrane-associated enzyme complex termed oligosaccharyltransferase (OST), which catalyzes the transfer of the high mannose oligosaccharide precursor Glc_3_Man_9_GlcNAc_2_ from a dolichol diphosphate-linked oligosaccharide donor to the side chain of an asparagine (Asn) residue [16]. The Asn residue appears as part of the consensus sequon Asn-X-Ser/Thr (X = any amino acid but proline). Following the transfer of the sugar chain by OST, the N-linked carbohydrate molecule undergoes sequential processing by several ER-localized enzymes, resulting in the appearance of a variety of structures, which serve as ligands for the ER protein folding and quality control machinery, a subject which will be discussed later and has been reviewed elsewhere [15,17,18]. 

OST is a multi-subunit membrane–protein complex composed of one catalytic subunit (either STT3A or STT3B) and at least six of the following accessory subunits in mammalian cells: Ribophorin 1 and 2 (RPN1, RPN2), DAD1, OST48, TMEM258, OST4, KCP2, DC2/OSTC, and either TUSC3/N33 or IAP/MAGT1 [19]. Each subunit spans the membrane and possesses a significant luminal domain. The OST and Sec61 complexes were found to be tightly bound to each other, probably in order to ensure the N-glycosylation of the consensus sequon as soon as the polypeptide emerges from the translocon to the ER lumen [20,21]. The immediate action of OST is crucial for the fate of the glycoproteins. It contributes to the solubility of nascent glycoproteins and directs them to the maturation and quality control machineries by creating recognition tags [22]. OST cryo-electron microscopy structures, which have recently been determined at atomic resolution, reveal its mode of association with the Sec61 translocon and with subunits recognizing the acceptor peptide during its appearance from the translocon [23].

The catalytic subunit STT3A associates with the protein translocation channel and acts in a co-translational fashion. STT3B post-translationally glycosylates specific sequons that are not glycosylated by STT3A [24]. OST48 and DAD1 have major roles in stabilizing both STT3A- and STT3B-containing OST complexes; hence, their depletion results in a severe and global hypoglycosylation phenotype that is directly comparable to that seen upon the depletion of both catalytic STT3 proteins. Similarly, OST4 is required to stabilize STT3 interactions [25]. KCP2 and DC2 are essential for the formation of the major STT3A-containing OST complex and its interaction with the translocon [26,27].

**Figure 1:**
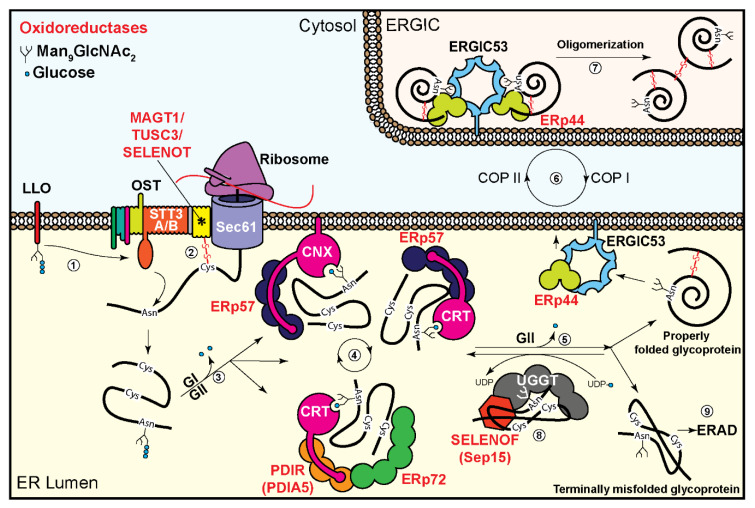
Glycoprotein translocation and folding in the endoplasmic reticulum (ER) in mammalian cells. (1) The oligosaccharide transferase (OST) complex co-translationally transfers the N-glycan precursor (Glc_3_Man_9_GlcNAc_2_) from the lipid-linked oligosaccharide (LLO) (Glc_3_Man_9_GlcNAc_2_-PP-Dolichol) to an asparagine residue in an N-glycosylation sequon of the glycoprotein during its translocation into the ER. (2) Sugar chain transfer to the glycoprotein is assisted by mixed disulfide bond formation between the glycoprotein and MAGT1 (IAP) or TUSC3 (N33) (in complex with the catalytic OST subunit STT3B), or with selenoprotein T (SELENOT) (in complex with the catalytic OST subunit STT3A). The names of other subunits of the large OST complex are omitted for simplicity. (3) The two terminal glucose residues are removed by Glucosidase I and II (GI and GII), respectively, allowing the glycoprotein to enter folding cycles by the ER chaperones calnexin (CNX) and calreticulin (CRT). (4) The oxidoreductase ERp57 interacts with CNX and CRT, assisting disulfide bond formation. Similar activity is observed by ERp72 indirectly interacting with CRT via PDIR. (5) The removal of the last glucose residue by GII results in the exit of the glycoprotein from the CNX cycle. (6) Properly folded glycoproteins are sent to the Golgi via the ER–Golgi intermediate compartment (ERGIC), mediated by a complex of ERGIC-53 and the oxidoreductase ERp44. (7) The ERGIC53/ERp44 complex is shuttled via COPII vesicles to the ERGIC, where it aids glycoprotein oligomerization. Unassembled subunits are shuttled back to the ER with ERp44 via COPI vesicles. (8) Misfolded glycoproteins are retained in the ER, where they are recognized by UDPGlc:glycoprotein glucosyltransferase (UGGT), with its 4 inactive Trxl domains in a complex with the seleno-oxidoreductase Sep15. UGGT reglucosylates the glycoprotein for re-entry into the CNX folding cycle. (9) Terminally misfolded glycoproteins are targeted to ER-associated degradation (ERAD). Oxidoreductases are marked in red.

OST complexes containing STT3B require one of two oxidoreductases (MAGT1 or TUSC3) as a subunit that is essential for OST function (Figure 1). MAGT1, also termed IAP, is an ER-localized thioredoxin homologue that associates with the STT3B catalytic subunit and is required for N-glycosylation at sites near cysteine residues. During transfer of the sugar chain, the N-X-S/T sequon must be in an extended strand conformation without a secondary structure. The majority of MAGT1 is present in the oxidized state and forms a transient mixed disulfide bond with a free thiol in the client glycoprotein, delaying intramolecular disulfide bond formation until STT3B glycosylates the acceptor sequon in the nascent polypeptide [28]. MAGT1 was also described as a Mg^2+^ transporter [29,30]. MAGT1-dependent glycosylation is sensitive to Mg^2+^ levels, and reduced Mg^2+^ affects immune cell function via the loss of specific glycoproteins, resulting in suboptimal T-cell activation. In healthy T and NK cells, MAGT1 is required for N-glycosylation and stabilizing key receptors, which are degraded in its absence. Defects in protein glycosylation and gene expression may lead to immune defects in inherited diseases due to MAGT1 deficiency [31,32,33]. Mutations in MAGT1 also cause glycosylation defects that lead to intellectual and developmental disability [34] and to X-linked immunodeficiency [35,36].

TUSC3, also called N33, is a homolog of MAGT1, and is enhanced in cases of MAGT1 deficiency as a result of a compensatory mechanism; however, each protein has a different tissue distribution in humans. MAGT1 and TUSC3 are orthologues of the *Saccharomyces cerevisiae* Ost3 and Ost6 proteins. Double knockout of Ost3p and Ost6p resulted in severe defects of N-glycosylation in yeast [37]. The same effect was observed in the absence of both MAGT1 and TUSC3 in mammalian cells [28]. Human MAGT1 and TUSC3 share 73% identity, possessing several highly conserved structural features. Each has an N-terminal signal peptide followed by a thioredoxin-like domain containing a bi-cysteine motif (CXXC), and a C-terminal domain composed of four membrane-spanning segments. The structures of the luminal domains of Ost6 and TUSC3 have been determined, revealing a thioredoxin fold with an active-site CXXC motif that is required for their function [38,39]. TUSC3 possesses a peptide-binding groove adjacent to the active-site cysteine pair, which binds peptides in opposite orientations. This allows TUSC3 control of sequon recognition and glycosylation efficiency through sequence-specific interactions with polypeptide segments of glycoproteins during co-translational translocation. Similarly to MAGT1, the oxidized form of TUSC3 was proposed to form transient mixed disulfides with a glycoprotein client, in order to facilitate access of STT3B to the unmodified acceptor sequon. TUSC3 also has oxidoreductase-independent functions in the STT3B-containing OST complex, possibly involving substrate recognition [39]. TUSC3/N33 was initially described as a candidate tumor suppressor based on the location of its gene on the short arm of chromosome 8, a region frequently lost in various tumors [40]. TUSC3 knockdown affects the ER structure and stress response and leads to accelerated tumorigenesis in prostate cancer cells under specific conditions [41]. It also enhances the proliferation and migration of ovarian cancer cells in vitro [42]. TUSC3 interacts with the protein phosphatase 1 (PPPC1A), and similarly to MAGT1, it has been linked to Mg^2+^ transport, having an important role in learning processes and short- and long-term memory, with its abnormal function leading to mental retardation [29,43,44].

Site-directed mutagenesis analysis revealed that the oxidoreductase CXXC active-site motif of MAGT1 or TUSC3 is essential for the glycosylation of Lassa virus spike glycoprotein, which mediates viral entry into host cells [45]. Catalytic oxidoreductase activity of MAGT1 is also required for Dengue virus propagation, possibly through an effect on viral protein synthesis or folding [46]. These results provide insight into the importance of the OST oxidoreductase subunits for viral interactions with host glycoproteins, and it was suggested that they could emerge as novel antiviral drugs, although potential toxicity should be evaluated.

In addition to MAGT1 and TUSC3, another oxidoreductase, Selenoprotein T, has recently been found to associate with OST complexes containing STT3A [47,48]. Selenoprotein T is a selenocysteine-containing protein with a thioredoxin-like domain containing an active-site CXXU motif. It was proposed that Selenoprotein T might fulfill a function in STT3A-containing OST complexes similar to that of MAGT1 and TUSC3 in STT3B-containing OST (Table 1).

## 3. Oxidoreductases Associated with the Glycoprotein Folding Machinery

Following ER translocation and glycosylation, glycoproteins are subjected to the ER folding and quality control machineries [82]. Proper folding, assisted by molecular chaperones, is critical for achieving the native state and correct protein function. One of the important steps involved in molecular chaperoning is stabilizing the nascent protein using intramolecular or intermolecular disulfide bonds between exposed adjacent cysteine thiol residues [11].

Glycoproteins are subjected to two major folding pathways. One is a general pathway, which also occurs for non-glycosylated proteins, involving the ER HSP70 protein BiP together with PDI and other oxidoreductases. BiP is the most abundant chaperone in the ER and consists of an N-terminal nucleotide-binding domain (NBD) and a C-terminal substrate-binding domain (SBD), and is a peptide-dependent ATPase that can either increase or decrease the folding rate of protein ligands. The hydrolysis of ATP-bound BiP to ADP is accelerated by ER co-chaperones of the DnaJ family (ERdj proteins), allowing substrate proteins to attain their native conformation and permitting the substrates to assemble with other subunits. The BiP/DnaJ complex also promotes accessibility to other chaperones such as PDI family members that generate and rearrange paired disulfide bonds. In some cases, such as those of P5 and ERdj5, the oxidoreductases interact directly, but non-covalently, with BiP [49,81].

The second pathway is specific for glycoproteins and involves the ER lectin chaperones, calnexin (CNX), an integral membrane protein, and its soluble homologue calreticulin (CRT) [52,83]. These molecular chaperones have two main functional domains, a lectin domain for binding N-linked sugar chains of newly-synthesized glycoproteins, and a proline-rich domain or P-domain, which binds to unfolded protein motifs and is responsible for the interaction with oxidoreductases [52]. One well-studied oxidoreductase, which forms complexes with CNX and CRT, is ERp57 [53]. While the lectin chaperones assist in the proper folding, ERp57 is involved in the disulfide bond formation between proximal cysteines [54,55]. Glycoprotein interaction with this system is regulated by sugar chain processing. The initial removal of the two outermost glucose residues from the N-linked oligosaccharide precursor by glucosidase I (GI) and glucosidase II (GII), respectively, creates a monoglucosylated sugar chain that is recognized by CNX or CRT [84]. Dissociation of CNX or CRT from the glycoprotein is elicited by removal of the third mannose-linked glucose by GII, which can be re-added by UDPGlc:glycoprotein glucosyltransferase (UGGT), leading to re-association with the lectin chaperones [52,85]. This “CNX cycle” continues until proper folding of the glycoprotein is achieved, which prevents further recognition by the folding sensor UGGT, allowing exit to the Golgi (Figure 1). If proper folding cannot be achieved in a certain timeframe, the glycoprotein molecules are targeted to ERAD by retrotranslocation to the cytosol and degradation by the ubiquitin–proteasome pathway, as we will discuss later. Whereas the newly-made glycoproteins associate with CNX and CRT in a complex with ERp57, the dissociation of the substrate glycoprotein from ERp57 and from the chaperone lectins follows different kinetics, which are dependent upon its folding state. Misfolded glycoproteins quickly dissociate from ERp57, while their association with CNX is prolonged, and the opposite is true for properly folded ones, implicating a role of ERp57 in productive glycoprotein folding [51].

ERp57 contains four thioredoxin-like domains, although only two of them, located at the N- and C-terminal ends, contain CXXC catalytic motifs [52]. Structural analysis showed a strong similarity between ERp57 and another ER localized oxidoreductase, ERp72. ERp72 lacks the binding domain to CNX and CRT. However, another protein with a thioredoxin-like domain, PDIR (also called PDIA5), was found to interact with CRT and with ERp72. The thioredoxin-like domain of PDIR does not contain an active catalytic motif, which suggests that a ternary complex CRT-PDIR-ERp72 may function in glycoprotein folding and disulfide bond formation, with PDIR participating in substrate recognition [56].

Recent findings have also shown the presence of 4 tandem thioredoxin-like domains in the CNX cycle glucosyltransferase, UGGT, suggesting a mechanism for its function as a folding sensor [57]. These domains of UGGT do not carry a catalytic site, and therefore lack a direct role in disulfide bond formation, but expose hydrophobic stretches for recognition of unfolded glycoproteins [58]. UGGT associates with a small selenoprotein, Sep15, also called Selenof [59]. Sep15 contains a catalytically active thioredoxin-like domain, implying that it may have a role in oxidoreductase activity on the glycoprotein substrate. A role of Sep15 in protein quality control is suggested by its upregulation under induced accumulation of misfolded proteins. The exact function of Sep15 is unknown, but its association with UGGT indicates its possible role in either modulating UGGT enzyme activity or assessing the disulfide bonds of the UGGT substrates. Sep15 knockout mice showed increased levels of secreted non-functional immunoglobulins, suggesting that it might function as a quality control gatekeeper for misfolded glycoproteins [59].

A series of membrane-bound lectins, namely ERGIC-53, VIP36, and VIPL, cycle between the ER, the ER to Golgi intermediate compartment (ERGIC), and the Golgi, binding and carrying properly folded glycoproteins that exit the ER. These lectins bind high mannose N-glycans with broad specificity, but with little to no affinity to mannose-trimmed Man_5-6_GlcNAc_2_, which as we will see in the next section are found on misfolded glycoproteins bound for ERAD [68,86]. It was shown that ERGIC-53 interacts with the oxidoreductase ERp44 and may act together in assembly of multimeric glycoproteins [69,70] (Figure 1). Many secretory proteins undergo oligomerization via intermolecular disulfide bond formation before exit to the Golgi, with the aid of oxidoreductases. ERp44 is the main oxidoreductase involved in this process. ERp44 was shown to regulate the traffic and the assembly of oligomers of IgM subunits and other secretory proteins [71,72]. It is also responsible for the ER retention of the oxidases ERo1α and Prx4 [71,73]. ERp44 traffics with its substrates from the ER to the ERGIC compartment via COP II vesicles, and it reduces the substrate thiol groups that will be involved in oligomerization. ERp44 is recycled to its active state by ERo1α or Prx4 in the ERGIC. The shuttling of ERp44 between the ER and the ERGIC is dependent on the pH difference between these two compartments and is regulated by the presence of Zn^2+^ in the ERGIC [74]. ERp44 carries a KDEL-like motif for its retrieval to the ER via COP I vesicles.

In the following sections, we will analyze the fate of glycoproteins that do not fold properly and do not traffic to their normal destination.

## 4. Involvement of Oxidoreductases in Mannose Trimming of Unfolded Glycoproteins

**Figure 2:**
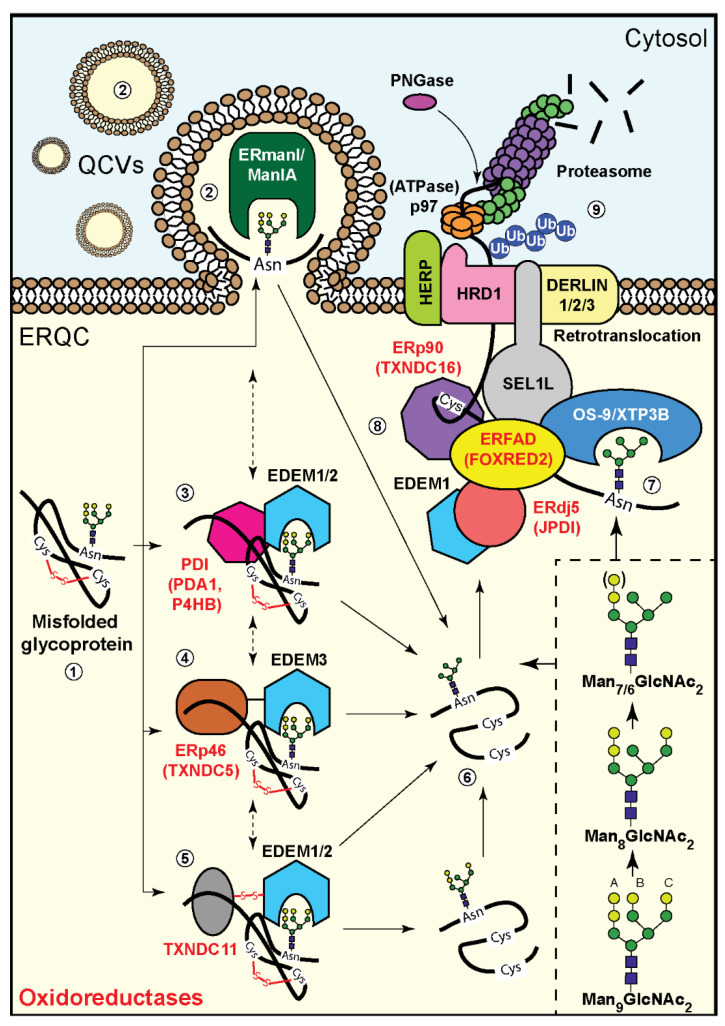
N-Glycan processing and ERAD in mammalian cells. (1) Misfolded glycoproteins carrying Man_9_GlcNAc_2_ are targeted to the ER-derived quality control compartment (ERQC), and their N-glycans are subjected to mannose trimming for their recognition by the ERAD machinery. A scheme on the right depicts mannose trimming of α1,2 mannose residues (light green circles, other mannoses are in dark green, and blue squares are GlcNAc). (2–5) This processing is performed sequentially or in parallel by the α1,2 mannosidases EDEM1-3, ManIA, or ERmanI. (2) ManIA or ERmanI (and possibly the EDEMs) reside in quality control vesicles (QCVs) and interact with the misfolded glycoproteins, likely at vesicle fusion sites. Several oxidoreductases interact with the EDEMs–(3) PDI (PDA1, P4HB) and (5) TXNDC11 associate with EDEM1 and 2, while TXNDC11 and (4) ERp46 (TXNDC5) associate with EDEM3. The associations of the oxidoreductases accelerate the mannose trimming activity of the EDEMs on misfolded glycoproteins, as shown so far for PDI and TXNDC11 with EDEM1/2 and ERp46 with EDEM3. (6,7) Trimming of three or all four α1,2 mannose residues results in glycoprotein molecules carrying Man_5-6_GlcNAc_2_ N-glycans, which bind to the OS-9/XTP3B lectins that interact with the ERAD complex. (7) OS-9 interacts with SEL1L, an adaptor protein for the E3 ubiquitin ligase HRD1, which associates with the membrane proteins Herp and the Derlins 1–3, among others. (8) OS-9 and SEL1L also interact with a complex of proteins containing Trxl domains, the non-catalytic ERp90 (TXNDC16), and the reductase ERdj5. ERp90 associates with the NAPDH-dependent reductase ERFAD. ERdj5 also interacts with EDEM1. These complexes lead to misfolded glycoprotein reduction, polyubiquitination, and retrotranslocation, with the help of the AAA ATPase p97 on the cytosolic side. (9) Peptide:N-glycosidase (PNGase) removes the N-glycans prior to deubiquitylation and degradation of the misfolded glycoproteins by the proteasomes. For simplicity, several ERAD components, such as p97 adaptors and proteasome shuttling factors, are not depicted.

One question that remained unanswered for many years was why the cell expends energy to assemble the large precursor N-linked oligosaccharide, only to then catalyze its sequential trimming. The modification of the N-glycan precursor turned out to be intimately linked to the folding, disulfide-bond formation, and complex assembly of glycoproteins. During the CNX folding cycle, N-glycan processing creates a code that reveals the folding status of each glycoprotein molecule, enabling continued folding attempts or targeting of the terminally misfolded glycoprotein for disposal. The code displayed by each structure produced by this trimming and its decoding by lectins has been the subject of research in many studies [63,66,76,77,87,88,89] and was reviewed in [15,18,90]. Several mannosidases are involved in the mannose trimming of the N-glycan precursor (Figure 2), which determines the fate of the glycoprotein, signaling whether it should be sent for degradation or exit to the Golgi and proceed to its final destination, as explained below.

N-glycans of misfolded glycoproteins, or of newly-made glycoproteins that fold very slowly, are more extensively trimmed than their properly folded counterparts, and removal of three or four α1,2-linked mannose residues signals their targeting to ERAD. Notably, removal of the terminal mannose residue from the A antenna prevents reglucosylation by UGGT1 and re-entry into the CNX cycle, ending any further protein folding attempts, while the removal of the terminal mannose residues from the B and C antennas allows binding of the N-glycan by the ERAD lectins OS-9 and XTP3-B, promoting degradation [77] (Figure 2). Receptor-mediated trafficking of properly folded glycoproteins out of the ER also relies on N-linked glycans. For example, as mentioned above, the lectin ERGIC-53 (along with VIPL and VIP36) help transfer correctly folded substrates to COPII vesicles for transport out of the ER to the Golgi. The shortened chains, Man_5-6_GlcNAc_2_, are recognized by the ubiquitination machinery-associated OS9 and XTP3-B, but not by the lectins that associate with properly folded glycoproteins trafficking to the Golgi that bind best to Man_8-9_GlcNAc_2_ [68]. Therefore, N-glycan trimming plays a crucial role in releasing substrates from the CNX cycle and targeting them to ERAD when proper folding is not achieved.

The enzyme that was initially implicated in removal of mannose residues, initiating the events that lead to misfolded glycoprotein export to ERAD, is ER mannosidase I (ERManI). ERManI has a preference for trimming the terminal mannose from the B antenna of the N-glycan, but was later found to be capable of removing all 4 α1,2-linked mannose residues [87,91]. ERManI was recently found to reside not in the ER, but in specialized quality control vesicles [92]. ERManI is assisted by the mannosidase activities of the ER-degradation-enhancing mannosidase-like proteins (EDEM1, 2, or 3). According to recent studies, ERManI has significant mannosidase activity on free N-glycans and denatured glycoproteins, but has only modest activity on native glycoproteins [63,93]. This difference is even more pronounced with EDEMs 1 and 2, which had little or no activity on free glycans, very modest activity on a native glycoprotein, and high activity on a denatured one [63]. In contrast to previous models suggesting general slow mannose trimming, this study revealed that misfolded or unfolded glycoproteins are subject to differentially faster trimming (and thus targeting to ERAD) than well-folded ones.

In addition to its recognition and trimming of N-glycans, EDEM1 can bind to misfolded proteins in a glycan-independent manner [94,95,96]. Furthermore, EDEM1 upregulation upon UPR induction can target substrate glycoproteins directly to ERAD, bypassing the requirement of mannose trimming to rapidly reduce the protein load in the ER [95].

Recent studies are starting to reveal how the EDEMs recognize unfolded or misfolded proteins, and again oxidoreductases are involved in this recognition. All 3 EDEMs were found to associate with oxidoreductases. EDEM1 and 2 associate with PDI, and also to a greater extent with an ER protein containing five thioredoxin-like domains, TXNDC11, which was implicated in ERAD [63,65] (Figure 2). In *Saccharomyces cerevisiae*, the association of the EDEM homolog Htm1p (Mnl1p) with PDI is a requirement for its mannosidase activity [60,64]. The interaction between Htm1p and PDI includes an intermolecular disulfide bond, which is essential for subsequent formation of a disulfide bond in the mannosidase homology domain of Htm1p, which in turn is in charge of substrate recognition and catalytic activity [97].

For EDEM1 and 2, the association of the oxidoreductases significantly enhanced the in vitro mannosidase activity on a native glycoprotein sample, but not on a denatured one [63]. This suggested that an oxidoreductase-mediated reduction and unfolding was required to expose hydrophobic domains of partially folded or misfolded substrate glycoprotein molecules in the native sample, whereas they were already exposed in the denatured sample. Exposure of the hydrophobic domains of the substrate would significantly enhance its recognition by EDEM1 and 2 [98]. EDEM1 and 2 showed little or no activity on free N-glycans. However, EDEM2 was recently shown to act on free N-glycans by forming a stable disulfide-bonded complex with TXNDC11, converting Man_9_GlcNAc_2_ to Man_8_GlcNAc_2_ (isomer B) in vitro [66], a similar preference as ERManI. Nevertheless, it was found that EDEM2 can also remove additional α1,2 mannose residues on glycoproteins [63].

TXNDC11 was also reported to be an interactor of EDEM3 [65], although the functional significance of this interaction it is still unknown. A recent study revealed that EDEM3 can associate stably with ERp46 (also called TXNDC5), another ER-resident oxidoreductase. This association was required for the mannosidase activity on misfolded substrate glycoproteins [67]. In vitro degradation of misfolded TCRα was reconstituted only when ERp46 had established a covalent interaction with EDEM3. This interaction, which depended on the redox activity of ERp46, involved formation of a disulfide bond between the cysteine residues of the ERp46 catalytic sites and the EDEM3 α-mannosidase domain. ERp46 does not directly change EDEM3 activity, and probably plays a role similar to that of TXNDC11 and PDI for EDEM1 and 2 activity, acting on the glycoprotein substrate [67]. Another study found a thiol-dependent substrate association of EDEM1 with the misfolded α1-antitrypsin variant Null Hong Kong (NHK), through a single Cys residue of the substrate, but this did not involve EDEM1 thiols. A similar thiol dependence was seen for association of the substrate with EDEM2 and 3 [99]. This suggests that it is the associated oxidoreductases, which possibly form mixed disulfides with the glycoprotein substrates.

Glycoproteins entering the Golgi undergo further mannose trimming by one or more of the resident α1,2 mannosidases. There are three “Golgi” α1,2 mannosidases—ManIA, IB, and IC. However, surprisingly, ManIA, which was thought to be a Golgi resident mannosidase, was recently found to localize to quality control vesicles, similarly to ERManI, and to be involved in glycoprotein quality control and targeting to ERAD [100] (Figure 2). It is unknown whether ManIA, IB, or IC activities are dependent on the folding status of the substrate glycoproteins or if any oxidoreductases associate with them, as in the case of the EDEMs. After α1,2 mannose trimming, natively folded glycoprotein molecules are further processed and targeted to their final destination, whereas misfolded glycoproteins are retrieved to the ER for ERAD or are recognized by quality control systems that operate in the Golgi complex and are delivered to lysosomes for degradation [101].

## 5. Oxidoreductases and Glycoprotein Targeting to ERAD

As discussed in the previous section, differential trimming of mannose residues from N-linked glycans determines whether glycoproteins are transported through the Golgi complex, or in the case of irreversibly misfolded glycoproteins, are retained in the ER and degraded by ERAD. This mechanism of protein surveillance is closely linked to redox and Ca^2+^ homeostasis. Ca^2+^ is necessary for the function of the major chaperones in the ER, including calnexin, calreticulin, BiP, and some PDIs. Inhibition of Ca^2+^ uptake into the ER causes ER stress due to inefficient protein folding and accumulation of unfolded or misfolded proteins. Consequently, maintenance of Ca^2+^ homeostasis is critically important for the functional integrity of the ER [102]. The main regulator of Ca^2+^ uptake into the ER is the sarco/endoplasmic reticulum calcium pump (SERCA), the activity of which is regulated by redox conditions (reviewed by Roscoe and Sevier in this Special Issue).

As we have seen, ER oxidoreductases are involved in the productive folding of newly synthesized glycoproteins, but they are also implicated in ERAD of misfolded glycoproteins. ERAD requires transport of a misfolded protein through the ER membrane to the cytosol, in a process called retrotranslocation. The mechanism involves recognition and segregation of terminally misfolded proteins from folding intermediates, targeting the former to membrane-bound ubiquitination complexes (the best-studied of which contains the HRD1 ubiquitin ligase), and finally degradation by cytosolic proteasomes (Figure 2). The HRD1 complex was suggested to be recruited by the homocysteine-induced ER protein (Herp) [103,104,105,106,107,108]. An important role in ERAD is played by p97 (Cdc48 in *Saccharomyces cerevisiae*), a member of the AAA (ATPase Associated with various cellular Activities) ATPase family. In conjunction with a large number of alternative cofactors and adaptors, p97 couples ATP hydrolysis to segregation of polypeptides from immobile cellular structures, such as protein assemblies, membranes, ribosomes, and chromatin. This often results in proteasomal degradation of the extracted polypeptides, and in ERAD p97 provides (in a complex with cofactors Ufd1 and Npl4) force for protein dislocation from the ER membrane to the cytosolic proteasomes [109,110,111,112,113]. P97 is recruited to the site of retrotranslocation through association with proteins present in the retrotranslocation complex, including the Derlins 1–3 and HRD1. These proteins each bear a p97 interacting motif, and the interactions with p97 allow the effective capture of substrates emerging from the proposed retrotranslocation channel [114].

There is evidence that the retrotranslocation process entails reduction of disulfide bonds of the substrate protein for unfolding, and several oxidoreductases have been implicated in this reduction and targeting to ERAD [115]. For the substrate recognition step, one non-catalytic member of the PDI-family, ERp90, interacts with misfolded proteins and associates with ERFAD, also known as FOXRED2, an NADPH-dependent reductase flavoprotein. In cells, ERFAD interacts with the ERAD luminal factors OS-9 and ERdj5 and the ER membrane protein SEL1L [78] (Figure 2). For glycoproteins, as explained above, mannose trimming allows recognition and binding to the lectins OS-9 and XTP3-B [76,77]. OS-9 associates with SEL1L, which in turn is in a complex with HRD1, thus targeting the glycoprotein for retrotranslocation [75]. One of the mannosidases involved in the trimming, EDEM1, has been found to associate with the oxidoreductase ERdj5. ERdj5, also known as JPDI (J-domain-containing PDI-like protein), functions as a reductase that reduces disulfides in misfolded ERAD substrates, such as α1-antitrypsin NHK, facilitating their retrotranslocation and degradation [79,80,81]. Biochemical analyses indicated that two thioredoxin domains that constitute the C-terminal cluster of ERdj5 form the reducing motif that interacts with EDEM1 and reduces disulfide bonds in EDEM1-associated glycoprotein substrates [79,80]. Through its J domain, ERdj5 also associates with BiP; ERdj5 appears to be crucial for ERAD, as ERdj5 knockout causes misfolded protein buildup and ER stress [50]. Nevertheless, the action of ERdj5 does not always lead to ERAD. Association of ERdj5 with the low-density lipoprotein receptor (LDLR) reduced non-native disulfides, allowing isomerization and productive folding [116]. Hence, ERdj5 acts as an ER reductase, both facilitating ERAD of misfolded proteins and catalyzing the folding of proteins, such as LDLR, that form obligatory transient non-native disulfides.

Another reductase that has recently been implicated in ERAD is TXNDC11. TXNDC11 was identified in screens involving gene trap mutagenesis and CRISPR/Cas9-mediated knockout in human haploid cells. It was found to be required for ERAD of various substrates, including unassembled CD3δ, TCRα, and mutant α1-antitrypsin NHK, but not for NHK-QQQ, a non-glycosylated version [65]. As mentioned before, TXNDC11 interacts with the EDEMs, enabling mannose trimming of their substrates. Therefore, the glycan processing appears to be coupled to the reduction of the disulfide bonds and targeting to retrotranslocation. PDI, which also interacts with EDEM1 and 2 [63], has likewise been implicated in reduction of glycoproteins for ERAD, in the turnover of Hedgehog, and in the degradation of mutant Akita pro-insulin in mammalian cells [61,62]. PDI is also required for ERAD of mutant carboxypeptidase Y (CPY*) in yeast [117].

## 6. Concluding Remarks

Studies in recent years have uncovered a tight cooperation between oxidoreductases and the glycoprotein processing machineries. This teamwork goes beyond disulfide bonding of cysteines in the proximity of sugar chains. Oxidoreductases interact with components of the machineries in charge of glycan addition, glycan processing, glycoprotein quality control, and ERAD targeting. They are involved in disulfide bond formation, the recognition of misfolded glycoproteins, and in accelerating the action of mannosidases that target the misfolded glycoproteins to ERAD). Oxidoreductases are also involved in disulfide reduction for isomerization of properly folded glycoproteins or before retrotranslocation and ERAD of misfolded glycoproteins. Nevertheless, we are only at the beginning of the discovery process, as the role of most of the multiple ER oxidoreductases or of their association with glycoprotein-specific machinery is still unknown.

Some of the questions that remain open are whether there are oxidoreductases acting on glycoproteins in post-ER compartments. Are there oxidoreductases functionally associated with the “Golgi” mannosidases IA, IB, and IC? Are other reductases involved in ERAD? What is the detailed mechanism of misfolded protein domain recognition by the oxidoreductases and is there an advantage when it involves mixed disulfide bonds? What are the mechanistic changes upon ER stress and oxidative stress? During oxidative stress, one could envisage a change in the balance of oxidases, favoring expression of those that consume peroxides. Upon ER stress, one could expect increased expression of both oxidoreductases that accelerate disulfide bond formation and reductases that increase disulfide bond reduction and facilitate ERAD to reduce ER protein load.

## Figures and Tables

**Table 1:** A list of oxidoreductases interacting with the glycoprotein processing and folding machinery. Glycoprotein biosynthesis in mammalian cells is separated into different processes, glycosylation, folding, N-glycan processing, ER–Golgi traffic, and ER associated degradation (ERAD). For each process, the specific proteins and their interacting oxidoreductase(s) are shown in the table. Respective thioredoxin-like (Trxl) domains and catalytic or non-catalytic motifs are shown.

Proteins	Interacting Oxidoreductases	Trx-Like Motifs
**Glycosylation (OST Complex)**		
STT3A	SELENOT (SELT) [47,48]	Active: Trxl1-CVSU
STT3B	MAGT1 (IAP) [28,29,30,31,32,33,34,35,36,45,46]	Active: Trxl1-CVVC
	TUSC3 (N33) [28,29,37,38,39,40,41,42,43,44,45]	Active: Trxl1-CSVC
**Protein Folding (Chaperones)**		
BiP *	PDI (PDA1, P4HB) [49]	Active: Trxl1,4-CGHC; Inactive: Trxl2,3
	P5 [49]	Active: Trxl1,2-CGHC
	ERdj5 (JPDI) [49,50]	Active: Trxl1-CPPC, Trxl2-CGPC, Trxl3-CHPC, Trxl4-CSHC
CNX	ERp57 [51,52,53,54,55]	Active: Trxl1,4-CGHC; Inactive: Trxl2,3
CRT	ERp57 [53,54,55]	
	ERp72 [56]	Active: Trxl1,2,5-CGHC; Inactive: Trxl3,4
	PDIR [56]	Active: Trxl2-CSMC, Trxl3-CGHC, Trxl4-CPHC; Inactive: Trxl1
**N-Glycan Processing (Glucose addition)**		
UGGT		Inactive: Trxl1 (aa 45–220), Trxl2 (aa 414–656), Trxl3 (aa 667–880), Trxl (aa 275–410 and 897–950) [57,58]
	SELENOF (Sep15) [59]	Active: Trxl1-CGU
**N-Glycan Processing (Mannose trimming)**		
EDEM1	P4HB (PDA1, PDI) [60,61,62,63,64]	
	TXNDC11 [63,65]	Active: Trxl1-CELC, Trxl5-CGFC; Inactive: Trxl2,3,4
EDEM2	PDI (PDA1, P4HB) [60,61,62,63,64]	
	TXNDC11 [63,65,66]	
EDEM3	TXNDC11 [65]	
	ERp46 (TXNDC5) [67]	Active: Trxl1,2,3-CGHC
**ER-Golgi trafficking and oligomerization**		
ERGIC-53	ERp44 (TXNDC4) [68,69,70,71,72,73,74]	Active: Trxl1-CRFS; Inactive: Trxl2,3
**ERAD (Retrotranslocation)**		
OS-9 and SEL1L [75,76,77,78]	ERp90 (TXNDC16)	Inactive: Trxl1-CX8C, Trxl2-CX9C, Trxl3-CX6C, Trxl4,5
	ERFAD (FOXRED2) **	NADPH-dependent reductase
EDEM1	ERdj5 (JPDI) [79,80,81]	

* BiP is not specific for glycoproteins. ** ERFAD does not have Trxl motifs.
